# Biodistribution and dosimetry of a single dose of albumin-binding ligand [^177^Lu]Lu-PSMA-ALB-56 in patients with mCRPC

**DOI:** 10.1007/s00259-020-05022-3

**Published:** 2020-09-19

**Authors:** Vasko Kramer, René Fernández, Wencke Lehnert, Luis David Jiménez-Franco, Cristian Soza-Ried, Elisabeth Eppard, Matias Ceballos, Marian Meckel, Martina Benešová, Christoph A. Umbricht, Andreas Kluge, Roger Schibli, Konstantin Zhernosekov, Horacio Amaral, Cristina Müller

**Affiliations:** 1 Center for Nuclear Medicine & PET/CT Positronmed, Julio Prado 714, 7501068 Providencia, Santiago Chile; 2 Positronpharma SA, 7500921 Providencia, Santiago Chile; 3 ABX-CRO, 01307 Dresden, Germany; 4 Department of Nuclear Medicine, University Medical Center Hamburg, 20251 Hamburg, Germany; 5 ITM Medical Isotopes GmbH, Munich, Germany; 6 Center for Radiopharmaceutical Sciences ETH-PSI-USZ, Paul Scherrer Institute, 5232 Villigen-PSI, Switzerland; 7 Department of Chemistry and Applied Biosciences, ETH Zurich, 8093 Zurich, Switzerland

**Keywords:** [^177^Lu]Lu-PSMA-ALB-56, PSMA-targeted radionuclide therapy, Albumin-binding PSMA radioligand, mCRPC, Prostate cancer, Dosimetry

## Abstract

**Introduction:**

PSMA-targeted radionuclide therapy with lutetium-177 has emerged as an effective treatment option for metastatic, castration-resistant prostate cancer (mCRPC). Recently, the concept of modifying PSMA radioligands with an albumin-binding entity was demonstrated as a promising measure to increase the tumor uptake in preclinical experiments. The aim of this study was to translate the concept to a clinical setting and evaluate the safety and dosimetry of [^177^Lu]Lu-PSMA-ALB-56, a novel PSMA radioligand with albumin-binding properties.

**Methods:**

Ten patients (71.8 ± 8.2 years) with mCRPC received an activity of 3360 ± 393 MBq (120–160 μg) [^177^Lu]Lu-PSMA-ALB-56 followed by whole-body SPECT/CT imaging over 7 days. Volumes of interest were defined on the SPECT/CT images for dosimetric evaluation for healthy tissue and tumor lesions. General safety and therapeutic efficacy were assessed by measuring blood biomarkers.

**Results:**

[^177^Lu]Lu-PSMA-ALB-56 was well tolerated, and no severe adverse events were observed. SPECT images revealed longer circulation of [^177^Lu]Lu-PSMA-ALB-56 in the blood with the highest uptake in tumor lesions at 48 h post injection. Compared with published data for other therapeutic PSMA radioligands (e.g. PSMA-617 and PSMA I&T), normalized absorbed doses of [^177^Lu]Lu-PSMA-ALB-56 were up to 2.3-fold higher in tumor lesions (6.64 ± 6.92 Gy/GBq) and similar in salivary glands (0.87 ± 0.43 Gy/GBq). Doses to the kidneys and red marrow (2.54 ± 0.94 Gy/GBq and 0.29 ± 0.07 Gy/GBq, respectively) were increased.

**Conclusion:**

Our data demonstrated that the concept of albumin-binding PSMA-radioligands is feasible and leads to increased tumor doses. After further optimization of the ligand design, the therapeutic outcomes may be improved for patients with prostate cancer.

**Electronic supplementary material:**

The online version of this article (10.1007/s00259-020-05022-3) contains supplementary material, which is available to authorized users.

## Introduction

Prostate cancer (PCa) is the most common type of cancer and the second leading cause of cancer death in men [1, 2]. The disease stage can range from slowly growing (i.e. localized disease) to rapidly progressive disease (i.e. metastasized cancer). In the latter case, the cancer cells may become resistant to androgen deprivation therapy (ADT) and chemotherapy, which makes it challenging to prevent further disease progression.

The prostate-specific membrane antigen (PSMA) is a transmembrane glycoprotein expressed in most PCa cells at considerably higher levels than in normal tissue. Importantly, the PSMA expression was shown to correlate with the stage of the disease [3, 4]. PSMA has, therefore, emerged as a promising target for molecular imaging and targeted radionuclide therapy of metastatic, castration-resistant PCa (mCRPC) [5, 6].

PSMA radioligands for positron emission tomography (PET) are used for the initial staging of high-risk tumors to identify sites of PCa recurrence and spread and to monitor therapy response. Additionally, the potential of PSMA radioligand therapy using β^−^- and α-emitting radionuclides and DOTA/DOTAGA-functionalized PSMA ligands (e.g. PSMA-617 and PSMA I&T) has been demonstrated in many clinical applications [7–11]. The overall positive therapeutic response to this treatment resulted in a Phase III clinical trial using [^177^Lu]Lu-PSMA-617 (VISION, NCT03511664) [12]. There is, however, still room for optimization, particularly with regard to the salivary gland uptake, which is high due to a not yet fully understood PSMA-unrelated uptake mechanism [13].

Years ago, the concept of using a small molecular weight albumin-binding entity to enhance the blood circulation time of radiopharmaceuticals was proposed [14–17]. Inspired by the promising example of folate radioconjugates, attempts to translate the “albumin-binder concept” to PSMA radioligands and other tumor-targeting agents were undertaken by several research groups [18].

At the Center for Radiopharmaceutical Sciences in Switzerland, the first glutamate-urea-lysine-based DOTA-functionalized PSMA ligands with a *p-*iodophenyl-entity were developed to enhance blood circulation [19]. Later, other research groups developed PSMA ligands based on the same functionalities, but with variable linker entities [20, 21]. Others used Evans blue as an albumin-binding moiety for modification of PSMA radioligands [22, 23]. In all cases, including a phosphoramidate-based PSMA radioligand [24], the tumor uptake was significantly increased as compared with the uptake of the respective control radioligand without an albumin-binding entity. These promising findings were, however, compromised by overly high blood activity levels and increased renal retention of these radioligands.

More recently, a PSMA ligand with a *p-*tolyl-entity was developed [14], in order to reduce the strong affinity to serum albumin and eventually optimize its pharmacokinetic profile [25]. Indeed, the tumor accumulation of [^177^Lu]Lu-PSMA-ALB-56 in mice was higher than for [^177^Lu]Lu-PSMA-617, while background retention was relatively low. The resulting efficacy of [^177^Lu]Lu-PSMA-ALB-56 for the treatment of PSMA-positive PC-3 PIP tumor-bearing mice was significantly improved as compared with that of the same activity of [^177^Lu]Lu-PSMA-617 [25].

The apparently beneficial clinical potential prompted us to initiate the clinical translation as proof-of-concept and to investigate the biodistribution and dosimetry of [^177^Lu]Lu-PSMA-ALB-56 in 10 patients with mCRPC. As an additional goal, the safety profile was assessed, based on blood parameters measured before and after treatment with [^177^Lu]Lu-PSMA-ALB-56. The results were discussed and compared with data reported for other relevant PSMA-targeting radiotherapeutics.

## Materials and methods

### Study design

The prospective study was designed including *n* = 10 patients to estimate tumor dosimetry, overall safety, and efficacy of a single dose of [^177^Lu]Lu-PSMA-ALB-56 in patients with mCRPC without options for conventional treatment. Based on follow-up data and individual evaluation, patients were offered the possibility to receive up to 3 additional cycles (with 10–13 weeks between cycles) of either [^177^Lu]Lu-PSMA-ALB-56 or [^177^Lu]Lu-PSMA-617. Study approval was obtained from the regional ethics committee board. All patients gave written informed consent, and all reported investigations were conducted in accordance with the Helsinki Declaration and with local regulations. Imaging visits and blood sampling were planned for day 0, 1, 2, and 7 post injection (p.i.) and clinical follow-up in week 4 and 10 after therapy.

### Subjects and treatment

From 01/2018 until 09/2018, ten mCRPC patients with disease progression under conventional treatment were included in the study (Table 1). Previous treatments included surgery, radiotherapy, first-line ADT, second-line ADT, and/or chemotherapy. Patients had PSMA-expressing lesions in the prostate bed (7/10), lymph nodes (LN) (8/10), bones (8/10), and soft tissue (2/10) evaluated by [^68^Ga]Ga-PSMA-11 (*n* = 2) or [^18^F]PSMA-1007 (*n* = 8) PET/CT scans within 1 week prior to the treatment. Blood biomarkers were evaluated at screening, at the day of treatment (baseline), and in week 4 and 10 p.i. (Table 1). [^177^Lu]Lu-PSMA-ALB-56 was prepared using non-carrier-added lutetium-177 (ITM Medical Isotopes GmbH, Germany; Supplementary material). An activity of 3360 ± 393 MBq (range, 2781–4252 MBq, 120–160 μg) was administered as a bolus injection followed by 10 mL saline.

**Table 1:** Patient characteristics

	Mean ± SD	Range	Normal
Patients			
Age (years)	71.8 ± 8.2	57–85	NA
ECOG baseline	0.6 ± 0.7	0–2.0	0
VAS baseline	1.1 ± 1.5	0–4.0	0
PSA (ng/mL)	153 ± 189	1.6–366	< 4.0
[^177^Lu]Lu-PSMA-ALB-56			
Activity (MBq)	3360 ± 393	2781–4252	NA
Mass (μg)	NA	120–160	NA
Blood biomarkers			
Erythrocytes (10^6^/μL)	3.9 ± 0.7	2.4–4.7	4.7–6.1
Hemoglobin (g/dL)	11.8 ± 2.1	7.5–14.1	14.0–18.0
Hematocrit (%)	35.2 ± 5.5	24.2–41.8	42.0–52.0
Leukocytes (10^3^/μL)	6.77 ± 3.14	2.8–12.9	4.5–11.0
Platelets (10^3^/μL)	243 ± 116	46–495	140–400
ESR (mm/h)	29 ± 16	12–54	1–15
Alkaline phosphate (U/L)	105 ± 53	57–204	40–130
GGT (U/L)	31 ± 31	10–89	15–73
LDH (U/L)	202 ± 39	135–255	0–250
Creatinine (mg/dL)	0.83 ± 0.11	0.63–0.97	0.7–1.2

*ECOG* eastern cooperative oncology group, *ESR* erythrocyte sedimentation rate, *GGT* gamma-glutamyl transferase, *LDH* lactate dehydrogenase, *PSA* prostate-specific antigen, *VAS* visual analog scale, *NA* not applicable

### Safety and efficacy

General safety, adverse events, and efficacy were assessed in week 4 and 10 after therapy by blood biomarkers according to Common Terminology Criteria for Adverse Events version 5.0 [26]. Alanine aminotransferase (ALT), alkaline phosphatase (ALP), aspartate aminotransferase (AST), blood creatinine (CRE), erythrocyte sedimentation rate (ESR), gamma-glutamyl transferase (GGT), lactate dehydrogenase (LDH), and total bilirubin (TBIL) were used as biomarkers of inflammation and kidney and liver function. Possible hematotoxicity was evaluated by hemoglobin, hematocrit, leukocytes, and platelets as biomarkers considering grade 3 and 4 anemia, leucopenia or thrombocytopenia as severe adverse events. Prostate-specific antigen (PSA) values were used as indicators for biochemical response or progression after treatment.

### SPECT/CT imaging

For each patient, whole-body (WB) SPECT/CT scans were acquired (three bed positions from the top of the head to the upper thighs; 90 projections and 25 s per projection) on a Symbia T2 scanner (Siemens Healthineers, Erlangen, Germany) at 1.5 ± 0.5 h, 6 ± 1 h, 24 ± 3 h, 48 ± 3 h, and at 7 days p.i. with a lutetium-177 reference-standard of approximately 10 MBq within the field of view. The scanner was equipped with a medium-energy low-penetration collimator. Three energy windows were acquired and used for further processing, a peak window of 20% width centered around the 208 keV energy peak and two adjacent corresponding lower and upper scatter energy windows of 10% width.

The SPECT images were stitched and quantitatively reconstructed using a commercial 3D ordered-subset expectation maximization (OSEM) algorithm (Flash 3D, Siemens Medical Solution, Germany) using 8 iterations and 9 subsets applying uniformity correction, CT-based attenuation correction, energy window-based scatter correction, and collimator-detector response modeling.

To yield quantitative images in units of Bq/mL, a calibration factor was determined from a phantom experiment using an IEC NEMA body phantom filled with 765 MBq lutetium-177 and applied to each patient SPECT dataset.

### Image processing and segmentation

Image processing and dosimetry analysis were performed using the QDOSE dosimetry software suite (ABX-CRO, Dresden, Germany). All SPECT images were co-registered to the low dose CT images. Kidneys (left and right), liver, spleen, salivary glands (left and right parotid and submandibular glands), urinary bladder content, eyes, total body, and up to 5 tumor lesions per patient were defined as source organs. Volumes of interest (VOIs) were segmented on either the SPECT or CT images, as described in the Supplementary material.

### Safety dosimetry

The time-activity curve (TAC) for the kidneys was calculated as the sum of the activities of left and right kidney. The TAC for the red marrow was calculated based on the activity concentration in the venous blood samples, obtained at 5 ± 2 min, 15 ± 5 min, 30 ± 5 min, 1.5 ± 0.5 h, 6 ± 1 h, 24 ± 3 h, 48 ± 3, and 7 days p.i., as follows [27]:

$$ {\mathrm{A}}_{\mathrm{red}\ \mathrm{marrow}}\ \left[\mathrm{MBq}\right]=\left({\mathrm{A}\mathrm{C}}_{\mathrm{blood}}\ \left[\mathrm{MBq}/\mathrm{mL}\right]\ast \mathrm{RMBLR}\ast 1500\ \mathrm{g}\right)/\left(1.05\frac{\mathrm{g}}{\mathrm{mL}}\right) $$

with A, activity; AC, activity concentration; and RMBLR, red marrow-to-blood activity concentration ratio and standard values for mass (1500 g) and density (1.05 g/mL). An RMBLR of 1.0 was applied as suggested for ^177^Lu-based peptide receptor radionuclide therapy (PRRT) [28].

All TACs were fitted depending on the degree of correlation to a mono- or bi-exponential function. The cumulated activity for each source organ and tumor was determined by calculating the area under the curve of the fitted TAC. The normalized cumulated activity (also time-integrated activity coefficient or residence time), was calculated for all source organs and tumors as the cumulated activity divided by the administered activity. The absorbed organ doses and effective dose calculations were performed using OLINDA/EXM 1.1 software [29] since it is the most widely used software available and has been used in most publications.

The absorbed doses to the salivary glands and eyes were determined using the spherical model [30] assuming organ masses of 25.0 g, 12.5 g, and 7.5 g for a single parotid gland, submandibular gland, and eyeball, respectively [31].

The results were used to determine the organs receiving the highest dose and the dose-limiting organs, using conservative absorbed dose limits of 28 Gy, 2 Gy, and 35 Gy for kidneys, red marrow, and salivary glands, respectively (EANM procedure guidelines) [32].

### Tumor dosimetry

Tumors were assumed to be spherical, and their volumes were calculated with sphere diameters based on the average of the two longest diameters in the axial view on contrast-enhanced CT images. Additionally, tumor masses were calculated with either a density of 1.06 g/cm^3^ for soft tissue lesions or 1.92 g/cm^3^ (same as cortical bone) for bone lesions (Supplementary material, Table S1) [33]. Absorbed dose calculations for soft tissue lesions were performed using the spherical model [30] implemented in QDOSE. Since this spherical model is only available for soft tissue, the absorbed doses to bone lesions were calculated using the spherical model in the freely available GUI version of IDAC-Dose 2.1, which allows dose calculations for tissues with different densities (including cortical bone) [34].

### Statistical analysis

All data were compared using the paired Wilcoxon test for paired data or Wilcoxon rank-sum test for independent samples. Two-sided *p* values of less than 0.05 (*p* < 0.05) were considered statistically significant. All analyses were performed using Stata software version 14.

## Results

### Pharmacokinetics of [^177^Lu]Lu-PSMA-ALB-56

**Fig. 1:**
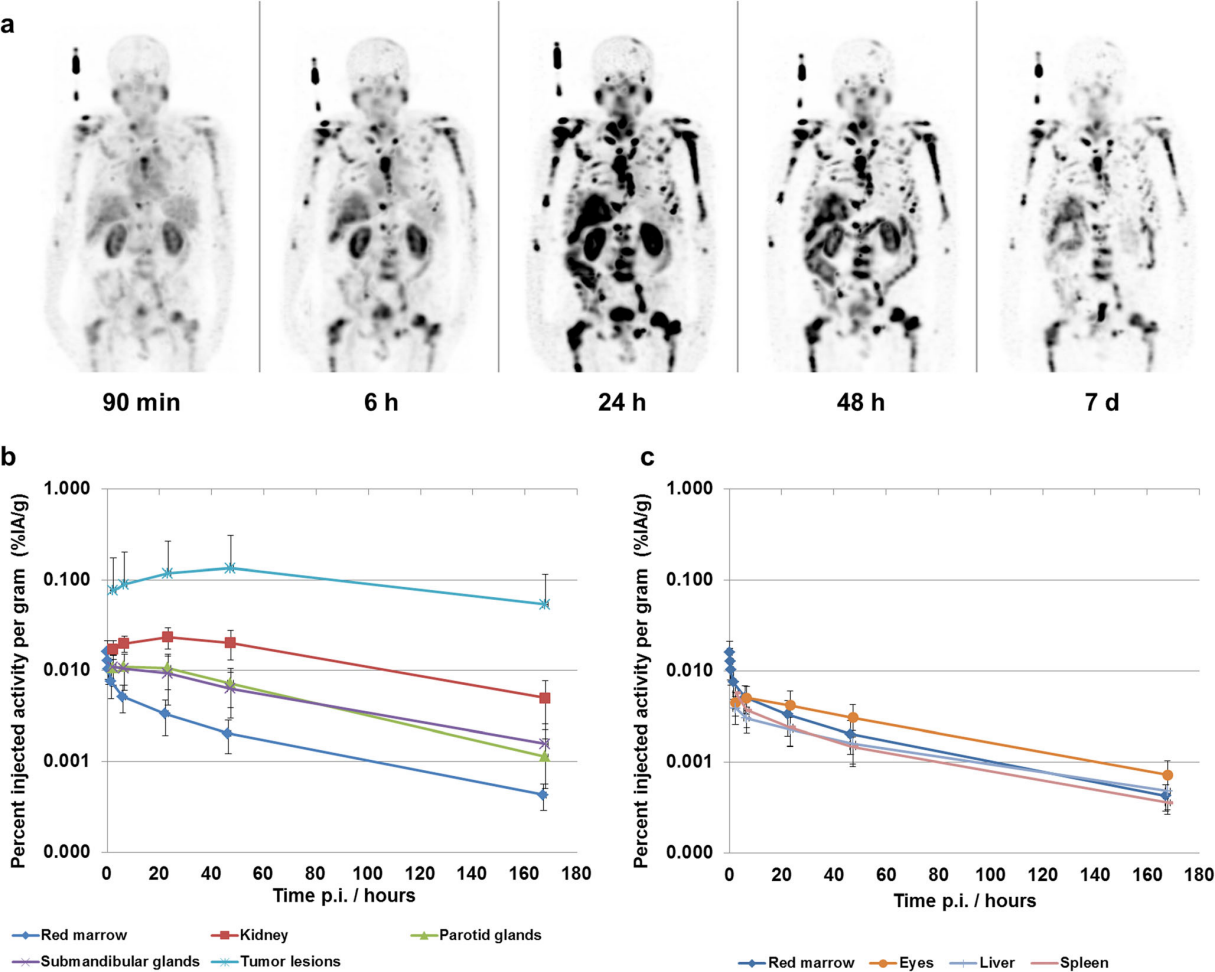
**a** Maximum-intensity projections of SPECT images of [^177^Lu]Lu-PSMA-ALB-56; **b** TACs for red marrow, kidneys, parotid glands, submandibular glands and tumor lesions expressed as percent injected activity per gram (% IA/g); **c** TACs for red marrow, eyes, liver and spleen (% IA/g)

Physiological uptake of [^177^Lu]Lu-PSMA-ALB-56 was observed in kidneys, parotid glands, submandibular glands, eyes, lacrimal glands, and blood (Fig. 1). The activity level in blood and red marrow decreased from 1.61 ± 0.53*10^−2^% IA/g at 5 min p.i. to 0.52 ± 0.17*10^−2^% IA/g at 6 h p.i. and further to 0.04 ± 0.01*10^−2^% IA/g after 7 days. The highest activity level in normal tissue was observed in the kidneys with peak uptake of 2.3 ± 0.6*10^−2^% IA/g at 24 h p.i., whereas uptake in the parotid and submandibular glands was significantly lower with 1.07 ± 0.46*10^−2^% IA/g and 0.93 ± 0.51*10^−2^% IA/g at 24 h p.i., respectively. In contrast to healthy organs, uptake of [^177^Lu]Lu-PSMA-ALB-56 in tumor lesions increased to 13.4 ± 17.4*10^−2^% IA/g at 48 h p.i.

### Organ dosimetry

The normalized absorbed doses for [^177^Lu]Lu-PSMA-ALB-56 were calculated for all individual patients (Table 2). In non-target tissues, the highest dose was observed for the kidneys with 2.54 ± 0.94 Gy/GBq (range 1.29–4.63 Gy/GBq), followed by parotid glands, submandibular glands, eyes, and red marrow with 0.87 ± 0.42 Gy/GBq (range 0.29–1.89 Gy/GBq), 0.87 ± 0.43 Gy/GBq (range 0.26–1.57 Gy/GBq), 0.36 ± 0.11 Gy/GBq (range 0.30–0.58 Gy/GBq), and 0.29 ± 0.07 Gy/GBq (range 0.15–0.40 Gy/GBq), respectively. Organ doses in other organs were significantly lower, and the whole-body effective dose was 0.20 ± 0.02 mSv/MBq (range 0.18–0.26 mSv/MBq).

**Table 2:** Normalized absorbed doses [Gy/GBq] for source organs and normalized effective doses [mSv/MBq] for all patients. NA: not applicable

Organ	Patient										Mean ± SD
	1	2	3	4	5	6	7	8	9	10	
Kidneys	2.95	2.09	2.43	2.32	1.38	1.29	2.69	4.63	3.03	2.55	2.54 ± 0.94
Right parotid gland	0.64	0.81	0.63	0.55	0.46	0.29	0.55	1.51	1.42	1.18	0.80 ± 0.42
Left parotid gland	0.77	0.85	0.72	0.63	0.56	0.45	1.08	1.89	1.26	1.10	0.93 ± 0.42
Right subm. gland	0.87	0.34	0.95	NA	0.31	0.26	1.14	1.22	1.18	1.37	0.85 ± 0.43
Left subm. gland	0.93	0.45	0.96	0.53	0.44	0.36	1.15	1.57	0.97	1.40	0.88 ± 0.42
Red marrow	0.24	0.27	0.30	0.35	0.27	0.15	0.28	0.40	0.37	0.26	0.29 ± 0.07
Eyes	0.38	0.20	0.32	0.42	0.30	0.30	0.48	0.58	0.33	0.32	0.36 ± 0.11
Liver	0.17	0.13	0.21	0.16	0.42	0.23	0.19	0.19	0.19	0.14	0.20 ± 0.08
Spleen	0.14	0.16	0.25	0.27	0.44	0.27	0.12	0.27	0.19	0.22	0.23 ± 0.09
Effective dose [mSv/MBq]	0.19	0.19	0.19	0.21	0.19	0.18	0.18	0.26	0.21	0.20	0.20 ± 0.02

### Tumor dosimetry

A total of 38 of 50 predefined tumor lesions were evaluated, whereas 12 of 50 lesions could not be clearly distinguished from surrounding structures. The tumor doses showed a strong variation between individual lesions (Table 3). In order to minimize the bias related to tumor size, only tumor lesions with a volume > 1.5 mL were considered. The overall normalized absorbed dose in tumor lesions > 1.5 mL was 6.64 ± 6.92 Gy/GBq (range: 0.42–122.7 Gy/GBq), and the tumor-to-kidneys absorbed dose ratio was 3.3 ± 2.8. The tumor doses in LNs and soft tissue lesions were significantly higher (12.7 ± 8.7 Gy/GBq; *n* = 10) as compared with tumor doses in bone lesions (3.6 ± 2.9 Gy/GBq; *n* = 17) (*p* < 0.05).

**Table 3:** Normalized absorbed doses [Gy/GBq] and tumor-to-kidney ratios for all lesions and for lesions > 1.5 ml (lesions < 1.5 mL are italicized). NE: lesions could not be distinguished from surrounding structures, NA: not applicable

Tumor lesion	Patient										Mean ± SD^a^
	1	2	3	4	5	6	7	8	9	10	
1	*3.60*	8.14*	*69.8*	7.77	NE	3.21*	*16.4*	*88.1*	6.51	3.82*	All lesions: 14.3 ± 25.2; > 1.5 mL: 6.64 ± 6.92
2	*9.52*	12.6	11.6*	6.62*	NE	2.62*	*14.0*	NE	10.9	7.69*	
3	2.99	123*	23.6	0.54*	0.36*	NE	NE	NE	28.8	1.55*	
4	5.13	6.97*	6.12*	7.15*	2.37*	NE	NE	16.2	NE	1.42*	
5	0.42*	NE	*13.8*	9.09*	1.55*	0.78*	NE	NE	4.87*	3.64	
Mean (all lesions)	4.33	37.6	25.0	6.23	1.43	2.21	15.2	52.2	12.8	3.62	NA
Tumor/kidney ratio^b^	1.50	18.0	10.3	2.70	1.00	1.70	5.70	11.3	4.20	1.40	5.80 ± 5.70
Tumor/sal. glands^b,c^	5.40	61.5	30.7	10.9	3.22	6.48	15.5	33.7	10.6	2.87	18.1 ± 18.7
Tumor/red marrow^b^	18.1	139	83.3	17.8	5.28	14.7	54.3	130	34.5	13.9	51.2 ± 49.9
Mean (> 1.5 mL)	2.85	9.79	23.6	6.23	1.43	2.21	NA	16.2	12.8	3.62	NA
Tumor/kidney ratio^b^	1.00	4.70	9.70	2.70	1.00	1.70	NA	3.50	4.22	1.40	3.3 ± 2.8
Tumor/sal. glands^b,c^	3.55	16.0	29.0	10.9	3.22	6.48	NA	10.5	10.6	2.87	10.3 ± 8.26
Tumor/red marrow^b^	11.9	36.2	78.7	17.8	5.28	14.7	NA	40.5	34.5	13.9	28.2 ± 22.6

^a^Mean ± SD for normalized tumor doses was calculated as mean across all individual lesions (*N* = 38 lesions in total; *N* = 27 lesions > 1.5 mL

^b^Tumor-to-organ ratios were calculated as mean value of individual patient–based tumor-to-organ ratios, based on mean tumor dose for a patient

^c^Tumor-to-salivary gland ratios were calculated based on mean dose across all four glands weighted equally

*Bone lesions were are identified by asterisks

### Safety and efficacy

**Fig. 2:**
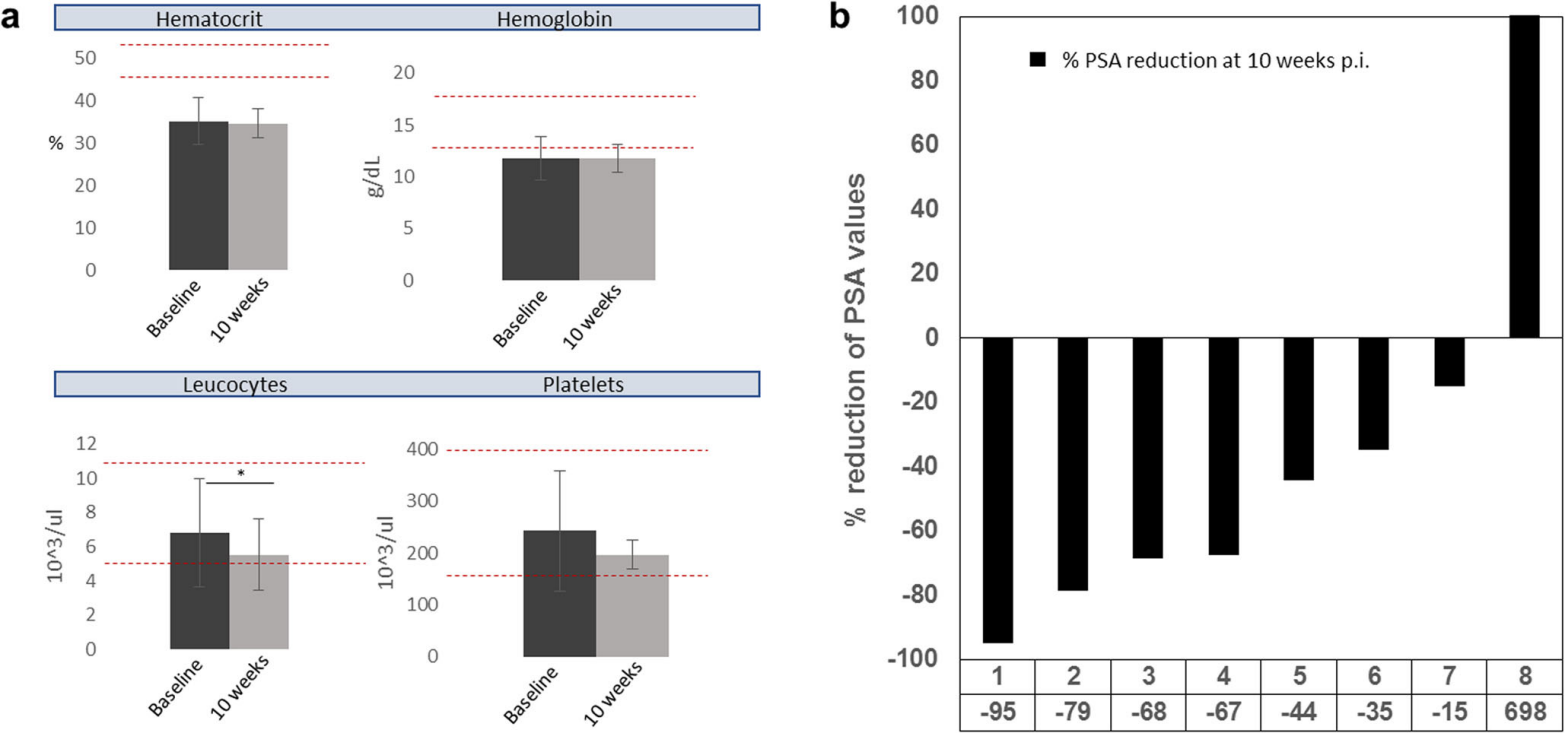
**a** Selected biomarkers for evaluation of hematotoxicity at baseline and 10 weeks after treatment. Red, dotted lines represent norm values. *p < 0.05. **b** Waterfall plot showing % changes of baseline PSA values

The treatment with [^177^Lu]Lu-PSMA-ALB-56 was well tolerated by all patients. There were no adverse effects, adverse drug reactions or significant changes in vital signs at the day of treatment. One patient, who was already in a critical condition before treatment, died of his disease in the following weeks. Another patient chose to withdraw from the study before the first follow-up visit. Thus, complete follow-up and blood biomarkers were obtained after 10.9 ± 2.4 weeks from 8/10 patients for evaluation (Fig. 2). No severe adverse drug reactions were observed. Two patients experienced grade 1 anemia (from normal baseline levels), and one patient experienced grade 2 anemia (from grade 1 at baseline). Four patients maintained their status, and one patient improved to grade 1 anemia (from grade 2 at baseline). Two patients with grade 1 anemia also developed leukocytopenia (grade 1 and grade 2, respectively). No significant changes (*p* < 0.05) of CRE, ALT, AST, ESR, ALP, GGT, LDH, and TBIL as markers of kidney and liver function were observed after treatment and within the period of observation (Supplementary material). A small but significant reduction of leukocytes was observed at 10.9 ± 2.4 weeks p.i. (range 8–14 weeks p.i.) (*p* < 0.05). The same trend was observed at 4 weeks p.i., but since, at that time, only data of 5/8 patients were available, it was not included in the overall analysis. Importantly, no patient experienced relevant xerostomia, fatigue, nausea, loss of appetite, nephrotoxicity, hepatotoxicity, or severe hematological toxicity.

In terms of efficacy, a significant reduction of PSA values was observed at 10.9 ± 2.4 weeks p.i. (range 8–9 weeks p.i.) (*p* < 0.05). On a patient-level, a reduction of > 50% of baseline PSA values in 4/9 patients (44%), a reduction of > 30% in 6/9 patients (67%), and any reduction of baseline PSA values in 7/9 patients (78%) were found. One patient (11%) showed biochemical progression, one patient (11%) died for whom we assume PSA progression, and one patient (11%) chose to retreat.

## Discussion

In spite of the great promise of preclinical results, experiences with the clinical translation of albumin-binding radiopharmaceuticals are scarce. Currently, there are only two studies reported in the literature, which investigated the “albumin binder concept” in human patients. Zhang et al. performed a clinical study, in which safety and dosimetry of Evans blue–modified DOTA-TATE were investigated [35]. Indeed, the tumor uptake of [^177^Lu]Lu-EB-DOTA-TATE was 6-fold enhanced compared with the uptake of [^177^Lu]Lu-DOTA-TATE; however, the dose to the kidneys and bone marrow was also increased [35]. Another clinical application referred to the investigation of [^177^Lu]Lu-EB-PSMA-617, which revealed increased tumor accumulation as compared with [^177^Lu]Lu-PSMA-617, but also higher uptake in kidneys and blood [36]. An advantage of using albumin-binding radiopharmaceuticals could, thus, be the possibility to implement a more convenient application protocol, which would require less frequent administration and/or lower activities per therapy cycle [36].

Our study was the first clinical application of [^177^Lu]Lu-PSMA-ALB-56, which is modified with a *p*-tolyl-entity as an albumin-binding entity. The aim of increasing the accumulation of activity in tumor lesions was successfully achieved. It resulted in a 1.4–2.3-fold higher absorbed dose to tumor lesions (6.64 Gy/GBq) as compared with published values for [^177^Lu]Lu-PSMA-617 (3.87 Gy/GBq [37], 4.60 Gy/GBq [38], and 2.80 Gy/GBq [39]) and [^177^Lu]Lu-PSMA I&T (3.30 Gy/GBq [40] and 3.20 Gy/GBq [41]), respectively.

Activity measurements of small tumor lesions can be biased due to partial volume effects, and the volume estimates are associated with higher uncertainties. Therefore, only lesions of > 1.5 ml were considered in our analysis, while inclusion of smaller lesions would have resulted in significantly higher dose estimates (*p* < 0.05) (factor of 2.15). As the details of tumor dosimetry methodology have not always been addressed in other publications, a direct comparison with other PSMA-based radioligands is challenging.

The mean absorbed dose to the salivary glands (0.86 Gy/GBq) was similar for [^177^Lu]Lu-PSMA-ALB-56 when compared with published data for [^177^Lu]Lu-PSMA-617 (Table 4) [37–39]. Only two studies performed dosimetry fully based on 3D WB SPECT/CT for [^177^Lu]Lu-PSMA-617, revealing higher (1.25 Gy/GBq) [36] or lower (0.51 Gy/GBq) [38] salivary gland doses. Together with a higher tumor absorbed dose, our data indicate a better tumor-to-salivary gland dose ratio for [^177^Lu]Lu-PSMA-ALB-56 (10.3 ± 8.3) as compared with [^177^Lu]Lu-PSMA-617 (2.7–9.0) [37, 38]. These findings were not in agreement with those reported for [^177^Lu]Lu-EB-PSMA-617 with a 6-fold increased salivary gland uptake as compared with [^177^Lu]Lu-PSMA-617 [36]. Comparisons have to be done cautiously, however, due to the differences in applied methodologies (e.g. planar imaging vs. SPECT; method of organ segmentation (activity and volume/mass); assumptions for organ masses; use of individualized parameters vs. literature parameters), which are often not reported in detail.

**Table 4:** Comparison of dosimetry data for different PSMA-targeting radiopharmaceuticals, currently under clinical investigation. Absorbed doses are presented as mean and SD values

Target dose	[^177^Lu]Lu-PSMA-ALB-56	[^177^Lu]Lu-EB-PSMA-617	[^177^Lu]Lu-PSMA-617			[^177^Lu]Lu-PSMA-I&T		[^177^Lu]Lu-J591
Method	WB SPECT/CT	WB SPECT/CT	WB Planar + Abdomen SPECT/CT	WB Planar	WB SPECT/CT	WB Planar	WB Planar	WB Planar
Kidneys (Gy/GBq)	2.54 (0.94)	2.38 (0.69)	0.61 (0.18)	0.60 (0.36)	0.39 (0.15)	0.80	0.72 (0.21)	1.41
Red marrow (Gy/GBq)	0.29^a^ (0.07)	0.054^b^ (0.006)	0.012^b^ (0.005)	0.042 (0.028)	0.11 (0.10)	0.03^a^	NA	0.32^b^
Salivary glands^c^ (Gy/GBq)	0.86 (0.42)	6.41 (1.40)	1.41 (0.53)	0.53 (0.20)	0.51 (0.40)	1.30	0.60 (0.27)	NA
Liver (Gy/GBq)	0.20 (0.08)	0.85 (0.24)	0.11 (0.06)	0.12 (0.06)	0.10 (0.05)	NA	0.12 (0.06)	2.10
Tumor (Gy/GBq)	6.64 (7.56)	NA	3.87^e^	2.80^f^	4.60^g^ (3.20)	3.30	3.20 (2.60)	24.3
Dose limiting organ^d^	Red marrow	Salivary glands	Salivary glands	Kidneys	Red marrow	Salivary glands	Kidneys	Red marrow
Maximum injectable activity (GBq)	6.89	5.46	24.8	46.7	18.2	26.9	38.9	6.25
Tumor dose at maximum injectable activity (Gy)	60.3	NA	96.1	131	83.6	88.8	124	151.9
Reference	this work	Zang et al. [36]	Delker et al. [37]	Scarpa et al. [39]	Violet et al. [38]	Baum et al. [40]	Okamoto et al. [41]	Vallabhajosula et al., [42]

*NA* not applicable

^a^Conservative estimation of RMBLR = 1.0 based on recommendations for ^177^Lu-PRRT [28]

^b^RMBLR of 0.36 is often used for ^177^Lu-PSMA dosimetry calculations [43]

^c^Tumor-to-salivary gland ratios were calculated based on mean dose across all four glands weighted equally

^d^Calculated with conservative absorbed dose limits of 28 Gy for kidneys, 2 Gy for red marrow, and 35 Gy for salivary glands, respectively [32]

^e^Calculated as mean absorbed dose across bone, LN and soft tissue lesions

^f^Calculated as mean absorbed dose across bone, LN and liver metastasis

^g^Calculated as mean absorbed dose across mean absorbed dose in bone and LN

The mean absorbed kidney dose of [^177^Lu]Lu-PSMA-ALB-56 (2.55 ± 0.93 Gy/GBq) was about 3.3-fold higher than reported values for [^177^Lu]Lu-PSMA-617 (0.60–0.88 Gy/GBq) resulting in a tumor-to-kidney dose ratio of 3.3 ± 2.8 compared with a previously reported dose ratio of ~ 5.1 for [^177^Lu]Lu-PSMA-617 [37]. As a general feature of albumin-binding radioligands, the blood activity levels of [^177^Lu]Lu-PSMA-ALB-56 were significantly higher than those of [^177^Lu]Lu-PSMA-617 and [^177^Lu]Lu-PSMA I&T. This was exemplified by blood activity data from a patient who received [^177^Lu]Lu-PSMA-ALB-56 and [^177^Lu]Lu-PSMA-617 as first and second cycle, respectively (Supplementary material, Fig. S2), which led to an increased estimated red marrow absorbed dose of 0.29 ± 0.07 Gy/GBq.

The red marrow was revealed as the dose-limiting organ for the application of [^177^Lu]Lu-PSMA-ALB-56 when considering a safe upper limit of 2 Gy. Considering the absence of any severe hematotoxicity in our study population, it is also possible that the red marrow dose was overestimated by assuming a conservative RMBLR of 1.0, which is in contrast to the study reporting on [^177^Lu]Lu-EB-PSMA-617, in which a factor of 0.32 was used [36]. The assumption of RMBLR = 1.0 was made by Baum et al. (2016) [40] for ^177^Lu-PSMA-I&T, while others used the earlier proposed RMBLR of 0.36 [37, 42, 44, 45]. In the latter case, the kidneys would have been the dose-limiting organ for [^177^Lu]Lu-PSMA-ALB-56, considering an absorbed dose limit of 28 Gy. Whether common measures such as amino acid infusion and/or pretreatment with diuretics (e.g. furosemide) would reduce kidney retention remains to be investigated [32].

Patients were offered to receive up to three additional therapy cycles. While the evaluation of long-term follow-up and efficacy for multiple cycles is beyond the scope of this article, it is worth mentioning that we did not observe any severe adverse drug reaction or toxicity in any of these patients after receiving the complete treatment.

As the therapeutic response to one cycle of 3.36 ± 0.39 GBq [^177^Lu]Lu-PSMA-ALB-56, we observed a decrease of the PSA values in 7/9 patients (78%), whereof 4/10 patients (44%) showed a decrease by > 50%. Hofman et al. enrolled 30 patients and evaluated them after one cycle of 7.5 GBq [^177^Lu]Lu-PSMA-617 [46]. In total, the PSA level was reduced in 77% of the patients, and the reduction was > 50% in 50% of these patients. In a larger, multicenter trial, Rhabar et al. found a reduction of > 50% in PSA in 40% of the patients and any response in PSA in 65% of the patients after one cycle of 5.9 GBq [^177^Lu]Lu-PSMA-617 [8]. The efficacy observed for [^177^Lu]Lu-PSMA-ALB-56, administered at 44–45% less activity, seemed, therefore, comparable with that of [^177^Lu]Lu-PSMA-617.

Certainly, larger cohorts of patients receiving multiple cycles would have to be investigated in order to draw final conclusions about the safe upper limit of applicable activity per therapy cycle and about the overall efficacy of ^177^Lu]Lu-PSMA-ALB-56.

The results of this study clearly indicate that further (pre)clinical research will be necessary to optimize the concept of albumin-binding PSMA radioligands, e.g. through the introduction of variable linker entities as recently exemplified by Deberle et al. [47].

## Conclusion

This prospective study demonstrated the specific tissue distribution profile of [^177^Lu]Lu-PSMA-ALB-56 in 10 patients with mCRPC and enabled dose estimations. The most interesting findings referred to the potentially increased tumor uptake and similar salivary gland accumulation, which would result in an increased tumor-to-salivary gland dose ratio as compared with [^177^Lu]Lu-PSMA-617. These promising clinical results are vital to understand the behavior of albumin-binding PSMA radioligands and for further optimization of the ligand design to reduce the risk of bone marrow and kidney toxicity. The interesting findings of this study will further be decisive for future research towards new administration protocols for albumin-binding PSMA radioligands.

## Electronic supplementary material

ESM 1 (DOCX 332 kb)

## Abbreviations

AC: activity concentration
ADT: androgen deprivation therapy
ALT: alanine aminotransferase
ALP: alkaline phosphatase
AST: aspartate aminotransferase
Bq: becquerel
CRE: creatinine
CT: computed tomography
ECOG: score eastern cooperative oncology group score
ESR: erythrocyte sedimentation rate
GGT: gamma-glutamyl transferase
Gy: gray
IA: injected activity
LDH: lactate dehydrogenase
LN: lymph nodes
mCRPC: metastatic, castration-resistant prostate cancer
PET: positron emission tomography
p.i.: post injection
PRRT: peptide receptor radionuclide therapy
PSA: prostate-specific antigen
PSMA: prostate-specific membrane antigen
RMBLR: red marrow-to-blood activity concentration ratio
SPECT: single photon emission computed tomography
Sv: Sievert
TAC: time-activity curve
TBIL: total bilirubin
VAS: visual analog scale
VOI: volume of interest
WB: whole body
