# Supplementary material for: Biodistribution and dosimetry of a single dose of albumin-binding ligand [177Lu]Lu-PSMA-ALB-56 in patients with mCRPC
Source: Eur J Nucl Med Mol Imaging. 2020 Sep 19;48(3):893–903. doi: 10.1007/s00259-020-05022-3 (PMC8036212; doi:10.1007/s00259-020-05022-3)
Supplement: Supplementary file 1 — (DOCX 332 kb) [file 259_2020_5022_MOESM1_ESM.docx]

**Supplementary Material**

**Biodistribution and dosimetry of a single dose of albumin-binding ligand [^177^Lu]Lu-PSMA-ALB-56 in patients with mCRPC**

Vasko Kramer^1,2*^, René Fernández^1^, Wencke Lehnert^3,4^, Luis David Jiménez-Franco^3^, Cristian Soza-Ried^1^, Elisabeth Eppard^2^, Matias Ceballos^1^, Marian Meckel^5^, Martina Benešová^6,7^, Christoph A. Umbricht^6^, Andreas Kluge^3^, Roger Schibli^6,7^, Konstantin Zhernosekov^5^, Horacio Amaral^1,2^ and Cristina Müller^6,7^

^1^ Center for Nuclear Medicine & PET/CT Positronmed, 7501068 Providencia, Santiago, Chile

^2^ Positronpharma SA, 7500921 Providencia, Santiago, Chile

^3^ ABX-CRO, 01307 Dresden, Germany

^4^ Department of Nuclear Medicine, University Medical Center Hamburg, 20251 Hamburg, Germany

^5^ ITM Medical Isotopes GmbH, Munich, Germany

^6^ Center for Radiopharmaceutical Sciences ETH-PSI-USZ, Paul Scherrer Institute, 5232 Villigen-PSI, Switzerland,

^7^ Department of Chemistry and Applied Biosciences, ETH Zurich, 8093 Zurich, Switzerland

^*^Corresponding author:

Vasko Kramer, PhD

Center of Nuclear Medicine & PET/CT Positronmed

Julio Prado 714

Santiago, Chile

Phone: +562 24205137

[vkramer@positronpharma.cl](mailto:vkramer@positronpharma.cl)

ORCID-ID: https://orcid.org/0000-0002-5285-6447

**EXTENDED MATERIALS AND METHODS**

**Radiochemistry**

Radiolabeling of [^177^Lu]Lu-PSMA-ALB-56 was performed using iQS-TS reagent kit for ^177^Lu-labeling and non-carrier-added lutetium-177 (both obtained from ITM Medical Isotopes GmbH, Munich, Germany). Briefly, labeling was performed by adding 160 μg (120.3 nmol) PSMA-ALB-56 dissolved in 1.0 mL ascorbate buffer pH=4.5 to 5.6 GBq (150 mCi) non-carrier-added lutetium-177 followed by heating to 95°C for 30 min. The reaction mixture was diluted with 0.9% sodium chloride and passed through a 0.22 µm sterile filter (Merck Millipore, Millex-GV SLGV033R). Quality control was carried out with an aliquot of the final product by radio-TLC (miniGITA, Elysia-Raytest, Straubenhardt, Germany) using silica 60 F254 TLC plates and two different solvents (solvent A: 0.1 M citrate buffer; solvent B: 1.0 M ammonium acetate buffer pH=4.5/methanol 1:1) and radio-HPLC (Elysia-Raytest, Straubenhardt, Germany; column: Phenomenex Onyx monolytic C18, 100x4.6 mm, flow: 1.5 mL/min, wave length 220 nm, solvent A: 0.1% TFA in water, solvent B: 0.1% TFA in acetonitrile, gradient: 0 min, 0% B; 0-20.0 min to 100% B; 20.0-25.0 min to 50% B). A radiochemical yield of ≥95% and a radiochemical purity ≥99% was obtained. An example of a radio-HPLC chromatogram is shown in figure S1.


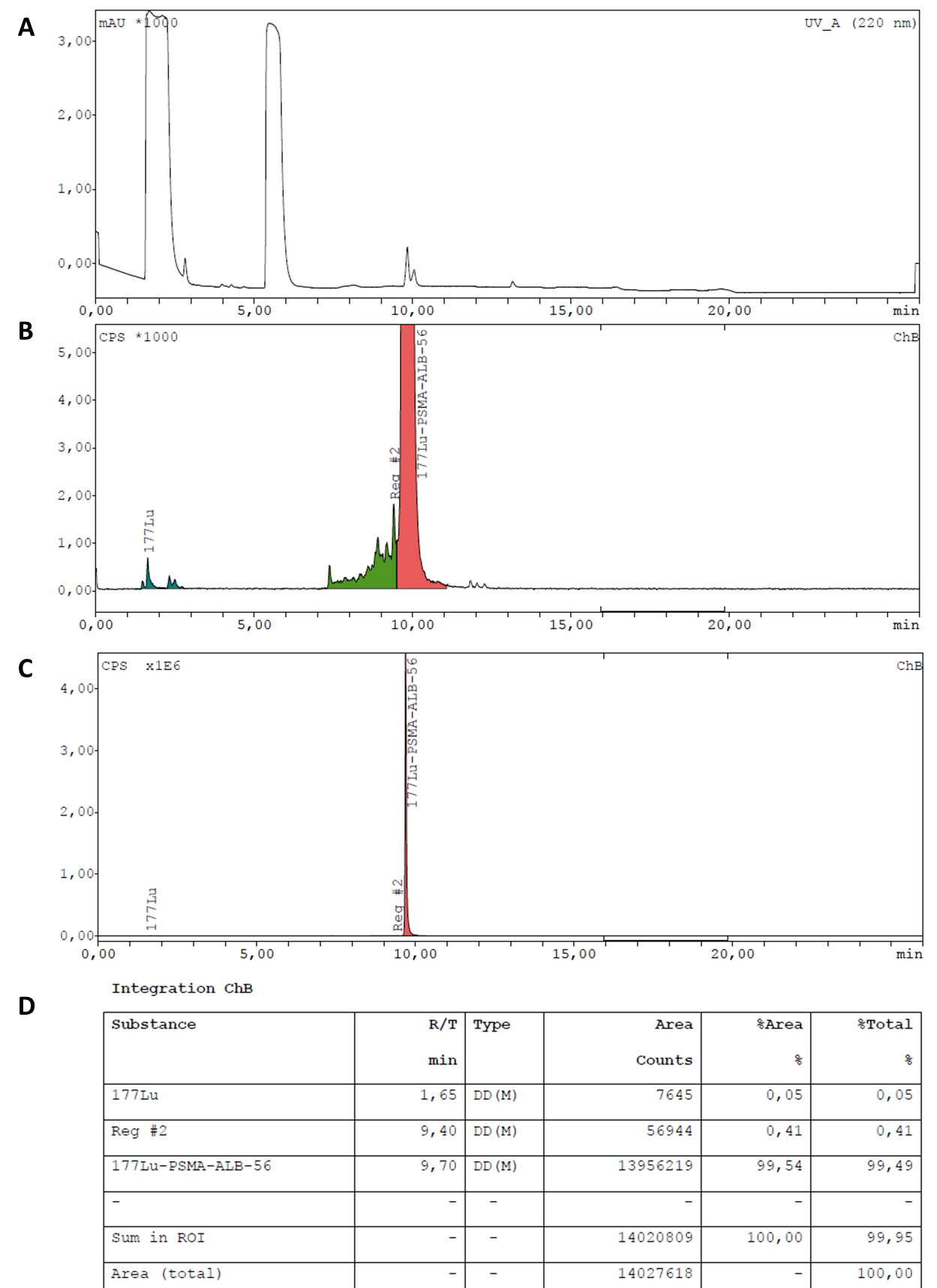


**Figure S1:** Example of radio-HPLC chromatogram of [^177^Lu]Lu-PSMA-ALB-56: A) UV-spectrum at 220 nm, B) Zoom of radio-chromatogram showing free lutetium-177 at 1.5 min, products from radiolysis from 7.0-9.5 min and [^177^Lu]Lu-PSMA-ALB-56 at 9.5-10.5 min, C) full scale radio-chromatogram, D) radiochemical purity.

**Image activity calibration**

To yield quantitative images in units of Bq/mL, a calibration factor was determined from phantom experiments using an IEC NEMA body phantom filled with 765 MBq ^177^Lu using the same acquisition and reconstruction parameters as later used for the patient scans. This was done prior to start of recruitment and repeated during the study. For the calibration, a large VOI excluding the cylindrical lung insert of the IEC NEMA body phantom was positioned inside the homogeneous background keeping some distance to the boundaries of the phantom. The total counts and volume for that VOI were calculated and a calibration factor (CF) was calculated according to the following formula:

$$CF \left[ \frac{Bq/ml}{cps/ml} \right]= \frac{Activity concentration [Bq/ml]}{\frac{Total Counts}{Nr. of projections*Time per projection \left[ s \right]*VOI Volume [ml]}}$$

For each patient scan, an additional ^177^Lu reference source was included in the field of view for quality control purposes, i.e. to assess the stability of the scanner in general and to check the proper scaling/calibration of the patient images within the dosimetry software. No adjustments to the calibration factor were made based on this source.

**Organ Segmentation**

The overall criteria for the VOI segmentation was to include all activity from the organ or tumor, without including activity from neighboring tissue. For some organs with good visibility on the CT, such as the liver or kidneys, initial semi-automatic segmentation was performed using the CT. The CT VOI was convolved with a Gaussian function (~ 5 mm full width at half maximum (FWHM) to account for the spillout due to the limited resolution of the SPECT. A threshold-based segmentation on the SPECT image was used with varying thresholds for the other organs. If required the VOIs, either based on CT or SPECT images, were manually adjusted to include all organ activity and exclude activity from neighboring structures. The volumes of interest (VOIs) of all segmented organs were then copied onto all other time points and, if necessary, adapted to calculate the time activity curves (TACs) for all organs.

**Tumor Dosimetry**

Tumor masses were calculated by multiplying the tumor volumes with either a density of 1.06 g/cm3 for soft tissue lesions or 1.92 g/cm3 for bone lesions. The estimated tumor masses are displayed in Table S1.

**Table S1:** Tumor masses**,** bone lesions are marked in yellow, NE: lesions could not be distinguished from surrounding structures, NA: not applicable.

|  | **Patient** | | | | | | | | | |
| --- | --- | --- | --- | --- | --- | --- | --- | --- | --- | --- |
| Lesion | **1** | **2** | **3** | **4** | **5** | **6** | **7** | **8** | **9** | **10** |
| **1** | 1.00 | 1.53 | 1.37 | 8.67 | NE | 25.81 | 0.74 | 0.09 | 2.73 | 4.52 |
| **2** | 0.70 | 5.14 | 2.21 | 37.79 | NE | 14.78 | 1.22 | NE | 34.21 | 29.95 |
| **3** | 7.47 | 0.13 | 2.73 | 69.29 | 133.36 | NE | NA | NE | 3.24 | 4.12 |
| **4** | 2.20 | 16.67 | 1.16 | 50.92 | 17.67 | NE | NA | 57.62 | NE | 64.34 |
| **5** | 35.10 | NE | 0.74 | 16.67 | 97.85 | 46.90 | NA | NA | 20.91 | 19.05 |


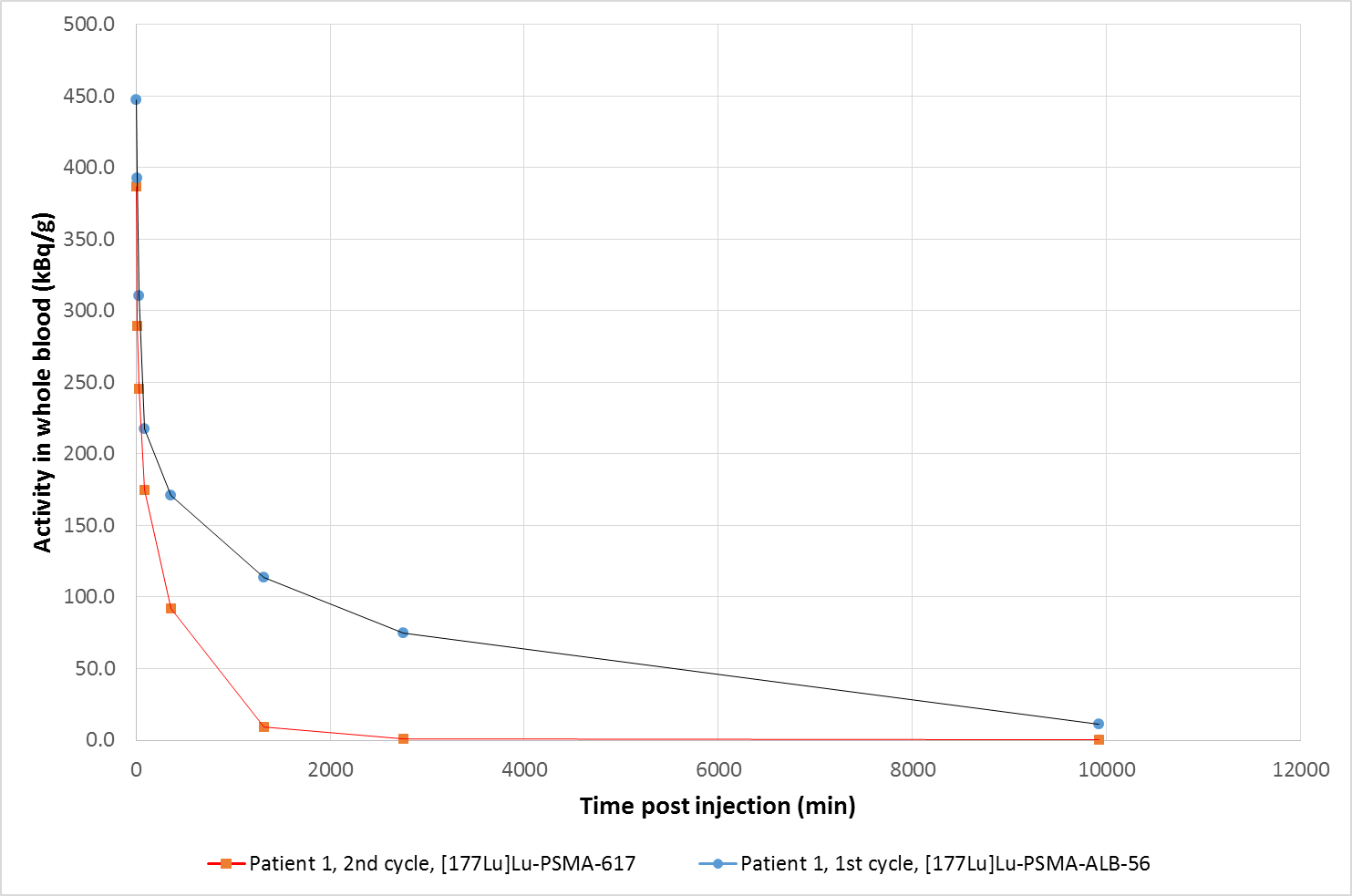


**Figure S2:** Time-activity-curves for whole blood measurements from first therapy cycle ([^177^Lu]Lu‑PSMA-ALB-56) and second therapy cycle ([^177^Lu]Lu-PSMA-617) for patient 1
